# Genomics on FHIR – a feasibility study to support a National Strategy for Genomic Medicine

**DOI:** 10.1038/s41525-025-00516-1

**Published:** 2025-07-29

**Authors:** Nina Haffer, Caroline Stellmach, Julian Sass, Michael R. Muzoora, Adam S. L. Graefe, Sylvia Thun, Carina N. Vorisek

**Affiliations:** Berlin Institute of Health at Charité - Universitätsmedizin Berlin, Berlin, Germany

**Keywords:** Computational biology and bioinformatics, Medical research

## Abstract

The German National Strategy for Genomic Medicine (genomDE) aims to integrate genome sequencing into standard healthcare. However, integrating genomics data from research and healthcare remains challenging. This study analyzed how the genomDE dataset could be mapped to international standards: the Genomics Reporting Fast Healthcare Interoperability Resources® (FHIR®) Implementation Guide (IG) 2.0.0, the Global Alliance for Genomics and Health (GA4GH)’s Phenopacket Schema, and the German national molecular genomics report IG of the Medical Informatics Initiative (MII). Sample FHIR® bundles and necessary search queries were created and validated. Most dataset elements could be represented using existing FHIR profiles, while unmapped elements were addressed through profiling and extensions. The study highlights that the genomDE dataset can largely be mapped to existing international standards, with the potential to extend these standards to accommodate missing elements, thereby improving genomic data interoperability in healthcare.

## Introduction

Genomic medicine has the potential to transform healthcare by integrating patients’ genomic information into clinical care^1^. It offers the possibility of a previously unprecedented level of precision in diagnosis and treatment of patients, particularly in oncology and rare diseases^2–5^. The genomDE initiative spearheads the idea of increasing the use of genomic analysis in patient care. It aims to establish a nationwide data infrastructure that bridges the gap between healthcare provision and research.6 The goal is to enhance the understanding of diseases and their subtypes at both clinical and pathophysiological levels. This integrated approach may facilitate more comprehensive analyses and foster deeper diagnostic capabilities and personalized therapeutic discovery^6,7^. Cross-entity feasibility within the genomDE infrastructure is of importance, with data provided by the German Familial Breast and Ovarian Cancer Consortium and other participating networks.

As genomDE connects specialized centers across Germany, aiming to create a nationwide platform for genomic medicine, institutions such as German Human Genome Archive (GHGA) play a central role in archiving and managing genomic data securely. The GHGA infrastructure is a federated network with GHGA data hubs located at seven sites throughout Germany. Thus, interoperability of said data is of high importance. The primary users include clinicians, researchers, and regulatory authorities. The system is designed to allow controlled access, ensuring that researchers can use anonymized or pseudonymized data while maintaining patient privacy and security.

genomDE not only focuses on its national strategy but also sets ground for international exchange of genomic data and therefore participates in the international consortia 1+ Million Genomes Initiative by the European Union. This initiative aims to provide a federated infrastructure for storage and analysis of genomic data. The federated approach, the sensitive nature of clinical data and the variety of genomic data format make the integration of interoperability a necessity. This is why the genomDE project prioritizes the concept of interoperability—the seamless exchange and functional use of information across diverse information technology (IT) systems. Furthermore, genomDE lays eyes on previously established interoperable data models and data sets to be able to exchange information nationally and internationally. Interoperability, however, is multifaceted, encompassing organizational, technical, semantic, and syntactic dimensions^7^. This feasibility study focuses on two key aspects: semantic and syntactic interoperability. Semantic interoperability is achieved through the adoption of international terminologies and ontologies, ensuring that the meaning of exchanged information is universally understood. For instance, the Sequence Variant Nomenclature (HGVS) provides a structured system for naming genetic variants at the DNA, RNA, and protein levels^8^. And Systematized Nomenclature of Medicine – Clinical Terms (SNOMED CT) is the largest medical terminology comprising more than 350.000 concepts to represent medical data. Syntactic interoperability pertains to the use of uniform data formats, facilitated by IT standards that dictate the structure of data.

The Health Level Seven (HL7) organization’s “Fast Healthcare Interoperability Resources” (FHIR®) standard was developed for healthcare purposes and is also gaining popularity in the domain of health research^9^. Previous work has explored the integration of genomics and clinical data using FHIR ®, such as vcf2fhir for genomic data conversion and SMART on FHIR Genomics for standardized applications, highlight the ongoing advancements in FHIR-based clinico-genomic integration^10–19^.

FHIR consolidates functionalities of its predecessors to offer a robust framework for data exchange, utilizing web technologies to enable comprehensive system integration. Within FHIR, resources serve as the building blocks, defining data elements, cardinalities, value sets, coding systems, and inter-resource references, which, for example, can link medication administration to a specific healthcare provider^20^. In addition, FHIR has been used for several use cases within genomic medicine: The National Institute of Health Cloud Platform Interoperability (NCPI) program is creating a federated ecosystem for genomic data that enhances researchers’ access to all data types^21^. The program’s external partners comprise three working groups addressing interoperability challenges specifically, one of them being the FHIR working group^22^. NCPI’s FHIR IG contains high level information and therefore was not included in our precise mappings. Furthermore, the Genomics Reporting IG by HL7’s Clinical Genomics Work Group^23^ and the GA4GH Phenopackets Schema represents significant advancements in standardizing the representation and exchange of genomic and phenotypic data^24^. Within the Genomics Reporting IG Version 2.0.0 fourteen FHIR profiles were developed enabling the representation of known and de novo variants, of simple and complex nature, somatic or germline origin^23^. It should be noted that a new version of the Genomics Reporting IG is now available that was not published yet at the time of the mapping process.

The specification also allows for deriving implications based on observed genomic characteristics regarding disease pathology, medication recommendations, diagnostics, and suitability for transplantation. The Phenopacket Schema ISO 4454:2022 defines a computable representation of clinical data to enhance the application of reusable analysis pipelines. There is a draft version of a FHIR representation of the Phenopacket Schema with an associated IG published on HL7 FHIR. As the Phenopacket provides a structured representation of phenotypic and genomic data that current FHIR Genomics standards do not yet fully cover and given its adoption by GA4GH and ongoing efforts to align it with FHIR, we anticipate that it will play a key role in future interoperability standards. Within the GA4GH Phenopacket Schema, a single person or bio sample is characterized in a Phenopacket and linked, among other things, with detailed phenotypic descriptions, genetic information, diagnoses, and medical treatments. A PhenotypicFeature represents the central element of the Phenopacket schema. Phenotypic characteristics such as symptoms, laboratory results, histopathological and radiological findings can be represented in a PhenotypicFeature with modification and qualification concepts^24,25^.

On a national level, the MII, formed by representatives from German university hospitals, research institutions, and the private sector have been developing the MII core dataset (KDS) based on FHIR and international terminologies such as SNOMED CT. The KDS is divided into basic and extension modules^26^. The extension modules represent specific medical disciplines such as molecular genetics, also called “MolGen Befund”, or in English: Molecular Genetic Findings Report^27^. The Extension module “MolGen Befund” or the Molecular Genetic Findings Report focuses on the structured representation of genetic characteristics with a FHIR IG^28^. genomDE played a key role in mapping and aligning the Molecular Genetic Findings Report dataset within the broader MII framework.

This study aims to assess the feasibility of mapping Phenotype sample data defined by genomDE to the HL7 FHIR framework, considering the foundational work of the already established international Genomic Reporting IG, GA4GH Phenopackets, and the national MII’s KDS. By examining and validating FHIR test data and query searches derived from molecular genetic findings, the study endeavors to enhance the precision and efficiency of international data exchange within genomic medicine, while considering national goals and requirements.

## Results

### FHIR resources

The sample data is mapped using the FHIR profiles of the KDS of the MII, the unreleased FHIR resources of the Phenopackets, and the HL7 FHIR Genomics Reporting.

### Mapping of data to HL7 FHIR

The Phenotype sample data from the GenomDE consortium included the data elements American College of Medical Genetics and Genomics (ACMG) criteria and classification according to 5-tier system which we regarded as equivalent classification system of sequence variants as defined by the American College of Medical Genetics and Genomics (see Table 1 and Supplementary Table 1)^29^.

**Table 1:** Mapping of data elements to the FHIR Resources and Profiles of the Medical Informatics Initiative Core Data Set and the Global Alliance for Genomics and Health Phenopacket Schema

Medical Informatics Initiative Core Data Set				Global Alliance for Genomics and Health Phenopacket Schema	
Nr	Data element	FHIR Ressource	FHIR Profile	Phenopacket Block	Phenopacket Element
1	**Submitter**	ServiceRequest.requester	MolGen Befund (Anforderung)	n.p.	n.p.
2	**Health status**	Condition	Diagnose (Condition)	Disease	Disease.term
3	**Diagnosis known at the time of test request**	ServiceRequest.reasonReference/DiagnosticReport.supportingInfo	MolGen Befund (Anforderung)	n.p.	n.p.
4	**Diagnosis based on genomic results**	Condition.evidence	Diagnose (Condition)	Diagnosis	Diagnosis.disease
5	**Phenotype according to HPO**	Condition.code; Observation.code	Diagnose (Condition); Symptome (Observation)	PhenotypicFeature	PhenotypicFeature.type
6	**Phenotype known at the time of test request**	ServiceRequest.reasonReference oder ServiceRequest.reasonCode	MolGen Befund (Anforderung)	(PhenotypicFeature)	(PhenotypicFeature.onset)
7	**Variant associated with a phenotype**	Observation.component:predicted-phenotype und/oder Observation.component:phenotypic-treatment-context	MolGen Befund (Diagnostische Implikation)/MolGen Befund (Therapeutische Implikation)	GenomicInterpretation	GenomicInterpretation.VariantInterpretation
8	**Diagnosis in ICD-10-GM**	Condition.code	Diagnose (Condition)	Disease	Disease.term
9	**Age/ birthdate**	Patient.birthDate	Person (Patient:in)	Individual	Individual.date_of_birth
10	**Age at diagnosis**	Condition.onsetDateTime	Diagnose (Condition)	Disease	Disease.onset
11	**ACMG Criteria**	Observation.component:clinical-significance	MolGen Befund (Diagnostische Implikation)	VariantInterpretation	VariantInterpretation.acmg_pathogenicity_classification
12	**OPS Code**	Procedure.code	Prozedur (Prozedur)	(Measurement)	(Measurement.assay)
13	**OMIM**	Condition.code	Diagnose (Condition)	Disease/Diagnosis	Disease.term/Diagnosis.disease
14	**pHGVS**	Observation.component:protein-hgvs	MolGen Befund (Variante)	VariationDescriptor	VariationDescriptor.Expression.value
15	**cHGVS**	Observation.component:coding-hgvs	MolGen Befund (Variante)	VariationDescriptor	VariationDescriptor.Expression.value
16	**Transcript (MANE)**	Observation.component:transcript-ref-seq	MolGen Befund (Variante)	n.p.	n.p.
17	**Classifikation (5er-System)**	Observation.component:clinical-significance	MolGen Befund (Diagnostische Implikation)	VariantInterpretation	VariantInterpretation.acmg_pathogenicity_classification
18	**Mean Coverage**	n.p.		n.p.	n.p.
19	**Readlength**	n.p.		n.p.	n.p.
20	**Single-End (SE)/Paired-End (PE) sequencing technology Mapped Read Percentage**	n.p.		n.p.	n.p.
21	**Coverage/DP Strand-Bias**	n.p.		n.p.	n.p.
22	**Variant fraction**	n.p.		n.p.	n.p.
23	**homozygous/heterozygous**	Observation.component:allelic-state	MolGen Befund (Variante)	VariationDescriptor	VariationDescriptor.allelic_state
24	**De novo-status**	Observation.component:genomic-source-class	MolGen Befund (Variante)	n.p.	n.p.

### Mapping of data elements with a focus on the MII’s KDS

Sender The term ‘Sender’ refers to the person who initiates the order (usually the treating physician). This corresponds in FHIR to the ServiceRequest.requester, in case molecular genetic tests are requested. For this use case, the following resources could be referenced: Practitioner, Organization, and PractitionerRole. (Profile MII PR MolGen Request for Genetic Test: https://www.medizininformatik-initiative.de/fhir/ext/modul-molgen/StructureDefinition/anforderung-genetischer-test).

The health status can be described with the Condition resource. The Condition describes a state and the assessment of a specific aspect of a patient’s health status by a clinician.

Within the clinical workflow, the process starts with an initial request from a healthcare provider for a diagnostic test (e.g., genetic testing, imaging, or lab work). During the diagnostics phase, relevant data is collected and analyzed through tests, observations, and reports. Finally, this information leads to the final diagnosis, which is documented as a Condition in FHIR, representing the clinician’s assessment of the patient’s health status. If the diagnosis is known at the time of test request, it can be referenced with ServiceRequest.reasonReference and/or DiagnosticReport.supportingInfo. If the diagnosis results from the genomic findings, the molecular genetic report can be referenced as the basis for the diagnosis with Condition.evidence. (Profile Profile - Condition - Diagnosis Version 2.0.0-alpha3: https://www.medizininformatik-initiative.de/fhir/core/modul-diagnose/StructureDefinition/Diagnose).

The phenotype according to Human Phenotype Ontology (HPO) can be described with the Condition or Observation resource. In the context of molecular genetic findings, the specification of the phenotype follows the workflow: if a phenotype is known at the time of test request, it can be referenced via ServiceRequest.reasonReference or ServiceRequest.reasonCode to the Condition or Observation. If the found variant is associated with a phenotype, it can be coded with Observation.component:predicted-phenotype and/or Observation.component:phenotypic-treatment-context. The phenotype is then specified using the HPO coding system. The GA4GH Phenopacket (Version 2) FHIR Implementation Guide in development uses the canonical URL as URI for the HPO coding system, while HL7 Terminology (THO) specifies the official canonical URL as follows: http://human-phenotype-ontology.org.

In practice, if the data is available in HPO, it should be encoded directly within the resources. However, for electronic medical record systems that primarily use SNOMED CT, the corresponding data can either be automatically mapped to HPO codes, or, if the coding is consistent within a cohort, SNOMED CT may also be used within a Phenopacket for a Phenotypic Feature – as the necessary Ontology Class can include both SNOMED CT and HPO codes. Notably, many information systems already use HPO, though this depends on country-specific implementation specifications. HPO is particularly advantageous for capturing phenotypic abnormalities, as it provides a more comprehensive overview of phenotypic expressions and their interrelationships, thereby facilitating advanced algorithmic analysis^30^.

- (Profile MII PR MolGen Request for Genetic Test: https://www.medizininformatik-initiative.de/fhir/ext/modul-molgen/StructureDefinition/anforderung-genetischer-test).
- (Profile MII PR MolGen Therapeutic Implication: https://www.medizininformatik-initiative.de/fhir/ext/modul-molgen/StructureDefinition/therapeutische-implikation).

(Profile MII PR MolGen Diagnostic Implication: https://www.medizininformatik-initiative.de/fhir/ext/modul-molgen/StructureDefinition/diagnostische-implikation).

Diagnoses in ICD-10-GM Similar to the specification of health status, diagnoses are indicated with the Condition resource. The coding system used is the tenth version of the International Classification of Diseases (ICD-10-GM) adapted to the German health system. HL7 Germany publishes the official canonical URL, which is: http://fhir.de/CodeSystem/bfarm/icd-10-gm.

- (Profile Profile - Condition - Diagnosis Version 2.0.0-alpha3: https://www.medizininformatik-initiative.de/fhir/core/modul-diagnose/StructureDefinition/Diagnose).

Age or Year of Birth The birth date is an element in the Patient resource, Patient.birthDate. The age must be calculated at the time of the question based on the birth date.

Age of Onset = Symptom onset or Age at Diagnosis The age of onset can be captured using Condition.onsetDateTime or Condition.onsetPeriod. Additionally, there is an extension for Condition.onsetPeriod that allows the life phase of symptom onset or age at diagnosis to be mapped using SNOMED CT.

- (Profile SD_MII_Person_Patient Version 2.0.0-ballot2: https://www.medizininformatik-initiative.de/fhir/core/modul-person/StructureDefinition/Patient).

ACMG Criteria In the context of pathogenicity assessment of a variant found through molecular genetic tests, the Observation.component:clinical-significance can be used to name the ACMG criterion using the LOINC-coded ValueSet http://loinc.org/vs/LL4034-6.

- (Profile MII PR MolGen Diagnostic Implication: https://www.medizininformatik-initiative.de/fhir/ext/modul-molgen/StructureDefinition/diagnostische-implikation).

The Operations and Procedures Key (OPS) is used in the Procedure resource in the Procedure.code element. The official canonical URL is published by HL7 Germany and is: http://fhir.de/CodeSystem/bfarm/ops.

Get requests are presented in Table 2: The data element “Sender” is represented through the element ServiceRequest.requester. FHIR describes in the core specification a search parameter requester of type reference, which can be used to query the referenced resource^31^. Here, an example search query is presented that queries all available ServiceRequest resources and returns the Practitioner resources in the bundle using _include^32^. For health status, here is a search query that queries all resources through the Condition endpoint of a server. It is possible to further narrow down the search by linking additional search parameters of the Condition resource^33^. The phenotype according to HPO can be specified as the reason for a request and is not queryable by standard search parameters in this case. The MII publishes a SearchParameter resource, which enables the previously presented request on ServiceRequest^31^. When specifying the phenotype within Observation resources, the query can be done through the code search parameter^34^. Diagnoses in ICD-10-GM can be searched through the code search parameter of the Condition resource^35^. The same search parameter can be used to search for OMIM codes. Age is not part of the Patient resource and therefore not queryable. However, it is possible to search by birth date using birthdate^36^. Search parameters of type date can be used with variable precision and thus can also serve to search by birth year only^32^. The ACMG criteria and classification (5-tier system) data elements are captured through the MII or Genomics Reporting Profile Diagnostic Implication/Diagnostic Implication in Observation.component:clinical-significance. The component can be retrieved using the LOINC code 53037-8. Besides the search parameter component-code, as shown in the example query, component-code-value-concept can be used to search for specific ACMG criteria^36^. An example query that only searches for pathogenic variants would thus look like this:

**Table 2:** Get Requests

Data element	Request
Submitter	GET [base]/ServiceRequest?&_include=ServiceRequest:requester
Health status	GET [base]/Condition
Phenotype according to HPO	GET [base]/ServiceRequest?reason-code=http://human-phenotype-ontology.org%7CHP:0001518 GET [base]/Observation?code=http://human-phenotype-ontology.org%7CHP:0001518.
Diagosis according to ICD-10-GM	GET [base]/Condition?code=http://fhir.de/CodeSystem/bfarm/icd-10-gm%7CQ87.1
Age year of birth	GET [base]/Patient?birthdate=2021
ACMG Criteria	GET [base]/Observation?component-code=http://loinc.org%7C53037-8
OMIM	GET [base]/Condition?code=http://www.omim.org%7C122470
pHGVS	GET [base]/Observation?component-code=http://loinc.org%7C48005-3
cHGVS	GET [base]/Observation?component-code=http://loinc.org%7C48004-6
Transcript (MANE)	GET [base]/Observation?component-code=http://loinc.org%7C51958-7
Classification (5er-System)	GET [base]/Observation?component-code=http://loinc.org%7C53037-8
Homo-/heterozygous	GET [base]/Observation?component-code=http://loinc.org%7C53034-5
De-novo status	GET [base]/Observation?component-code=http://loinc.org%7C48002-0

**Fig. 1:**
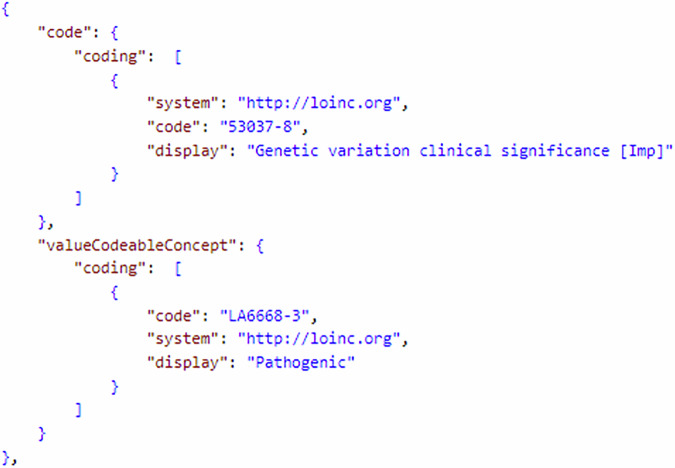
Screenshot of results of a query searching for pathogenic variants.

GET[base]/Observation?component-code-value-concept=http://loinc.org%7C530378$http://loinc.org%7CLA6668-3 (see Fig. 1).

Every additional specified search query for cHGVS, pHGVS, Transcript (MANE), homo-/heterozygous, and De novo status works as described for ACMG criteria via the respective LOINC code for the Observation.component and the search parameters component-code or component-code-value-concept.

## Discussion

This feasibility study has provided insight into the current capabilities and limitations of mapping genomDE’s predetermined data elements to FHIR resources specified in national and international FHIR IGs such as the MII’s KDS, HL7’s Genomics Reporting IG, and the GA4GH’s Phenopackets IG.

The integration of genomics and clinical data using HL7 FHIR has also been explored in previous work. GEMINI, a framework for integrating genomics and clinical data using FHIR to enable statistical analysis, demonstrating practical feasibility in a real-world healthcare setting, has been introduced in 2020^10^. In the United States, the eMERGE Network^11^ provides insights into the implementation challenges of genomics-electronic health record integration, emphasizing interoperability considerations within FHIR-based systems. Additionally, Dolin et al. introduced vcf2fhir, a utility that converts Variant Call Format (VCF) files into HL7 FHIR format, facilitating genomic data incorporation into EHRs^12^. Key genetic lab test elements have been mapped to HL7 FHIR specifications, ensuring compliance with professional reporting guidelines^13^. Similar advancements have also been published in South Korea, where Seong et al.^14^. implemented a FHIR-based quality information exchange system for clinical next-generation sequencing genomic testing. Additionally, SMART on FHIR Genomics^16^, facilitates standardized clinico-genomic applications by leveraging the SMART on FHIR platform. Lastly, considering the mCODE (Minimal Common Oncology Data Elements) framework and the Precision Oncology Core Data Model could provide valuable insights, particularly for cancer-related applications^18,19^.

We were able to map 19 out of 24 data elements to FHIR resource profiles in the MII’s KDS IGs. For five genomDE dataset elements, no corresponding FHIR modeling was found, highlighting a potential area for expansion in future versions. For the unmappable elements HL7 FHIR also supports open slicing^37^. The mapping process has demonstrated compatibility across FHIR profiles in both the MII KDS IGs and HL7’s Genomics Reporting IG V2.0.0. GenomDE dataset elements were mapped to those specified in four MII KDS modules’ IGs: the Diagnosis^38^, Procedure^39^, Person^40^, and MolGen Report modules^41^ were considered in this process. It is worth noting that the absence of the data element ‘patient age’ within the MII’s KDS; The MII specification only specifies the birthdate in the Patient resource. Therefore, a patient’s age at a particular point in time would have to be calculated and could potentially be documented using an Observation resource.

Additionally, the onset time of a disease is indicated in the *Condition* resource using Condition.onsetDateTime or Condition.onsetPeriod, from which the age at onset can be calculated. The MII’s Diagnosis profile includes a “Life Phase” extension with specific SNOMED CT codes, reflecting different stages of life.

The study encountered challenges in mapping the sequencing and variant metadata elements from the genomDE dataset to preexisting FHIR specifications. However, we identified modeling options and propose the use of Observation.component:region-coverage, Observation.component:allelic-read-depth, and Observation.component:sample-allelic-frequency within the *Observation* resource

We considered the ACMG variant classification system to be equivalent to the 5-tier classification data elements defined in the genomDE dataset. Should the stakeholders of the genomDE initiative wish to use an alternate classification system (not the ACMG), then the FHIR mapping would need to be adjusted accordingly.

Secondly, we found that 17 of the 24 dataset elements defined by the genomDE initiative could be mapped to profiles in the GA4GH Phenopacket IG V0.1.0. However, it should be noted that although GA4GH’s Phenopacket IG as well as the MII’s Molecular Genomics IG are both derivatives of HL7’s Genomics Reporting IG, these two specifications use a different version of the international FHIR specification, thus they are incompatible. As they are two of the few international FHIR specifications available we still mapped the dataset to both. Version discrepancies lead to incompatibilities in data element usage, ValueSets, and coding systems within the profiles. Consequently, it is not feasible to operate a FHIR server with both the current KDS Module MolGen/Genomics Reporting version and the Phenopacket FHIR IG simultaneously. Moreover, the Phenopacket FHIR Implementation Guide V0.1.0 does not publish a FHIR package, rendering it impossible to configure a FHIR server with the Conformance resources and test an implementation.

Most of genomDE’s proposed dataset elements can be structured and exchanged using FHIR. For data elements for which no preexisting model was available, it is feasible to extend existing specifications with elaborately modeled profiles and resources; however, this requires significant resources. For genomDE to establish a FHIR-based data exchange infrastructure, the participating sites must implement the MII KDS specifications and be able to provide data in the described formats. This data could then be transferred to a central repository supporting the FHIR RESTful API, which would offer validation through the FHIR API and possess the necessary properties, such as terminologies. In conclusion, while most genomDE data elements are transferable via FHIR in a structured manner, the study reveals a need for the creation and adoption of extended profiles and resources to accommodate unmapped elements. It would be beneficial for future work if more than two moleculargenetic reports were available for generating test data. Such advancements will pave the way for a more comprehensive and integrated genomic data infrastructure, enhancing the potential for personalized medicine and more effective healthcare delivery.

## Methods

### Mapping activities

The data elements to be represented were determined by the stakeholders of genomDE, based on use cases such as genetic testing for hereditary cancer syndromes and rare disease diagnostics. It is important to note that data elements describing the mutational patterns and clonal heterogeneity of somatic tumors are outside the scope of this study. As a result, only the relevant data elements were mapped onto FHIR profiles and resources from the MII KDS extension module ‘Molekulargenetischer Befundbericht’ (Version 1.0.0), the Genomics Reporting Working Group (Version 2.0.0), and the GA4GH Phenopacket Schema (Version 2.0).

The mapping was conducted by four experts: a biotechnologist, a biochemist, a medical computer scientist, and a clinician. The different backgrounds are aimed to bring a diverse perspective to the mapping and thus achieve the best possible results.

### Testing the generated data on the FHIR server

For testing purposes, a HAPI FHIR JPA Server was utilized to load the MII KDS and Genomics Reporting IG conformance resources. The FHIR resources for the two test reports were transferred to the endpoint of the FHIR server via a HTTP POST request.

### FHIR queries

For the genomDE data elements, exemplary FHIR Search queries were written, enabling the querying of data within the test findings.

## Supplementary information

Supplementary Information

## Data Availability

No datasets were generated or analysed during the current study.

## Abbreviations

ACMG: American College of Medical Genetics and Genomics
cHGVS: Human Genome Variation Society
FHIR®: Fast Healthcare Interoperability Resources®
GA4GH: Global Alliance for Genomics and Health
genomDE: German National Strategy for Genomic Medicine
GHGA: German Human Genome Archive
HGVS: Sequence Variant Nomenclature
HL7: Health Level Seven
HPO: Human Phenotype Ontology
IG: Implementation Guide
ICD: International Statistical Classification of Diseases and Related Health Problems
IT: Information Technology
KDS: MII Core Dataset (Kern Datensatz)
OMIM: Online Mendelian Inheritance in Man
MANE: Matched Annotation between NCBI and EBI
mCODE: Minimal Common Oncology Data Elements
MII: Medical Informatics Initiative
NCPI: National Institute of Health Cloud Platform Interoperability
OPS: Operationen-und Prozedurenschlüssel
pHGVS: Human Genome Variation Society
SNOMED CT: Systematized Nomenclature of Medicine – Clinical Terms

## Code availability

No specific code was developed or used, as the metadata was directly mapped to the FHIR standard.
