# Supplementary material for: Genomics on FHIR – a feasibility study to support a National Strategy for Genomic Medicine
Source: NPJ Genom Med. 2025 Jul 29;10:57. doi: 10.1038/s41525-025-00516-1 (PMC12307893; doi:10.1038/s41525-025-00516-1)
Supplement: Supplementary file 1 — Supplementary Information [file 41525_2025_516_MOESM1_ESM.pdf]

Supplementary Table 1 I Mapping of data elements to the FHIR Resources, Profiles and ValueSets of the Medical Informatics Inititiative Core Data Set and the Global Alliance for Genomics and Health Phenopacket Schema.

Nr	Data element	MII KDS FHIR Ressource	MII KDS FHIR Profil	MII KDS ValueSets	Phenopacket Block	Phenopacket Element	Phenopacket VS
1	**Submitter**	ServiceRequest.requester	MolGen Befund (Anforderung)		n.p.	n.p.	
2	**Health status**	Condition	Diagnose (Condition)		Disease	Disease.term	
3	**Diagnosis known at the time of test request**	ServiceRequest.reasonReference / DiagnosticReport.supportingInfo	MolGen Befund (Anforderung)		n.p.	n.p.	
4	**Diagnosis based on genomic results**	Condition.evidence	Diagnose (Condition)		Diagnosis	Diagnosis.disease	
5	**Phenotype according to HPO**	Condition.code; Observation.code	Diagnose (Condition); Symptome (Observation)		PhenotypicFeature	PhenotypicFeature.type	
6	**Phenotype known at the time of test request**	ServiceRequest.reasonReference oder ServiceRequest.reasonCode	MolGen Befund (Anforderung)		(PhenotypicFeature)	(PhenotypicFeature.onset)	

7	**Variant associated with a phenotype**	Observation.component:predicted-phenotype und/oder Observation.component:phenotypic-treatment-context	MolGen Befund (Diagnostische Implikation) / MolGen Befund (Therapeutische Implikation)		GenomicInterpretation	GenomicInterpretation.VariantInterpretation	
8	**Diagnosis in ICD-10-GM**	Condition.code	Diagnose (Condition)	http://fhir.de/ValueSet/bfarm/icd-10-gm (required)	Disease	Disease.term	
9	**Age/ birthdate**	Patient.birthDate	Person (Patient:in)		Individual	Individual.date_of_birth	
10	**Age at diagnosis**	Condition.onsetDateTime	Diagnose (Condition)		Disease	Disease.onset	
11	**ACMG Criteria**	Observation.component:clinical-significance	MolGen Befund (Diagnostische Implikation)	http://loinc.org/vs/LL4034-6 (extensible)	VariantInterpretation	VariantInterpretation.acmg_pathogenicity_classification	https://phenopacket-schema.readthedocs.io/en/latest/variant-interpretation.html#rstacmgpathogenicityclassification (required)
12	**OPS Code**	Procedure.code	Prozedur (Prozedur)	http://fhir.de/ValueSet/bfarm/ops (required)	(Measurement)	(Measurement.assay)	
13	**OMIM**	Condition.code	Diagnose (Condition)		Disease / Diagnosis	Disease.term / Diagnosis.disease	

14	**pHGVS**	Observation.component:protein-hgvs	MolGen Befund (Variante)	http://hl7.org/fhir/uv/genomics-reporting/ValueSet/hgvs-vs (required)	VariationDescriptor	VariationDescriptor.Expression.value	
15	**cHGVS**	Observation.component:coding-hgvs	MolGen Befund (Variante)	http://hl7.org/fhir/uv/genomics-reporting/ValueSet/hgvs-vs (required)	VariationDescriptor	VariationDescriptor.Expression.value	
16	**Transcript (MANE)**	Observation.component:transcript-ref-seq	MolGen Befund (Variante)		n.p.	n.p.	
17	**Classifikation (5er-System)**	Observation.component:clinical-significance	MolGen Befund (Diagnostische Implikation)	http://loinc.org/vs/LL4034-6 (extensible)	VariantInterpretation	VariantInterpretation.acmg_pathogenicity_classification	
18	**Mean Coverage**	n.p.			n.p.	n.p.	
19	**Readlength**	n.p.			n.p.	n.p.	
20	**Single-End (SE)/Paired-End (PE) sequencing technolog**	n.p.			n.p.	n.p.	

	**y Mapped Read Percentage**						
21	**Coverage/ DP Strand-Bias**	n.p.			n.p.	n.p.	
22	**Variant fraction**	n.p.			n.p.	n.p.	
23	**homozygous/heterozygous**	Observation.component:allelic-state	MolGen Befund (Variante)	http://loinc.org/vs/LL381-5 (extensible)	VariationDescriptor	VariationDescriptor.allelic_state	https://phenopacket-schema.readthedocs.io/en/latest/variant.html#allelic-state (recommended)
24	**De novo-status**	Observation.component:genomic-source-class	MolGen Befund (Variante)	http://loinc.org/vs/LL378-1 (extensible)	n.p.	n.p.	
